# Mechanisms of the antinociceptive action of (−) Epicatechin obtained from the hydroalcoholic fraction of *Combretum leprosum* Mart & Eic in rodents

**DOI:** 10.1186/1423-0127-19-68

**Published:** 2012-07-25

**Authors:** Luciano da Silva Lopes, Rosemarie Brandin Marques, Heliana Barros Fernandes, Sergio da Silva Pereira, Mariane CC Ayres, Mariana Helena Chaves, Fernanda RC Almeida

**Affiliations:** 1 NPPM – Medicinal Plants Research Center, Health Sciences Center, Federal University of Piauí (UFPI), Av. Nossa Senhora de Fátima s/n, 64049-550, Teresina, PI, Brazil; 2 Department of Chemistry, Natural Sciences Center/UFPI, Teresina, PI, Brazil

**Keywords:** (−) epicatechin, Antinociception, Combretum leprosum, Glutamate, Serotonin and opioids

## Abstract

**Background:**

The mechanisms of the antinociceptive activity of (−) epicatechin (EPI), a compound isolated from the hydroalcoholic fraction of *Combreum leprosum* Mart & Eicher.

**Methods:**

were assessed in the model of chemical nociception induced by glutamate (20 μmol/paw). To evaluate the mechanisms involved, the animals , male Swiss mice (25-30 g), received EPI (50 mg/kg p.o.) after pretreatment with naloxone (2 mg/kg s.c. opioid antagonist), glibenclamide (2 mg/kg s.c. antagonist K + channels sensitive to ATP), ketanserin (0.3 mg/kg s.c. antagonist of receptor 5-HT_2A_), yoimbine (0.15 mg/kg s.c. α2 adrenergic receptor antagonist), pindolol (1 mg/kg s.c. 5-HT1_a_/1_b_ receptor antagonist), atropine (0.1 mg/kg s.c. muscarinic antagonist) and caffeine (3 mg/kg s.c. adenosine receptor antagonist), ondansetron (0.5 mg/kg s.c. for 5-HT_3_ receptor) and L-arginine (600 mg/kg i.p.).

**Results:**

The antinociceptive effect of EPI was reversed by pretreatment with naloxone and glibenclamide, ketanserin, yoimbine, atropine and pindolol, which demonstrates the involvement of opioid receptors and potassium channels sensitive to ATP, the serotoninergic (receptor 5HT_1A_ and 5HT_2A_), adrenergic (receptor alpha 2) and cholinergic (muscarinic receptor) systems in the activities that were observed. The effects of EPI, however, were not reversed by pretreatment with caffeine, L-arginine or ondansetron, which shows that there is no involvement of 5HT_3_ receptors or the purinergic and nitrergic systems in the antinociceptive effect of EPI. In the Open Field and Rotarod test, EPI had no significant effect, which shows that there was no central nervous system depressant or muscle relaxant effect on the results.

**Conclusions:**

This study demonstrates that the antinociceptive activity of EPI in the glutamate model involves the participation of the opioid system, serotonin, adrenergic and cholinergic.

## Background

**Figure 1:**
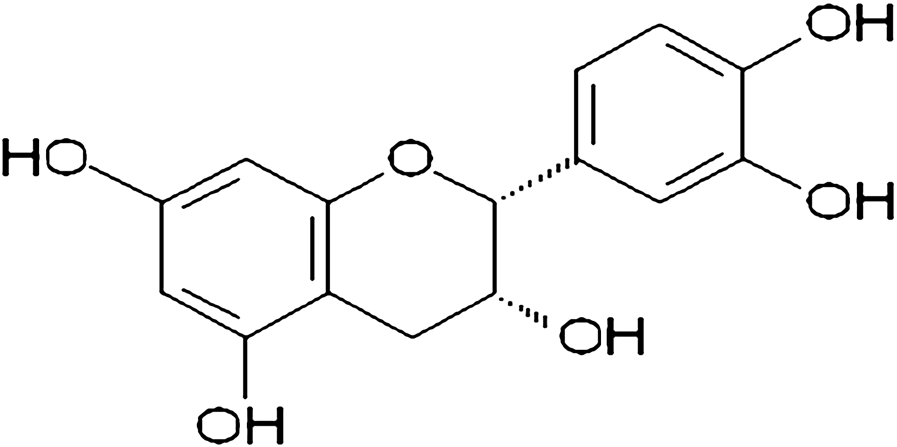
Chemical struture of (−) Epicatechin.

One of the most noteworthy chemical constituents of *Combretum leprosum* Mart & Eic is (−) epicatechin (Figure 1). Is a flavanol belonging to the catechin family and is commonly present in monomeric and/or oligomeric forms in green teas and red wine. A flavonoid known mainly for its antioxidant, cardioprotective, anti-inflammatory and antineoplastic properties [1-6].

However, previous studies have shown that this class of substances has the ability to block some subtypes of sodium channels including neuronal cells in vitro, which could be related to possible antinociceptive properties [7-9]. Other studies have shown that polyphenolic compounds have inhibitory properties on the formation of nitric oxide, an important gauge of the phenomena involved in nociception [10]. It is interesting to note that hardly any studies in the literature have assessed the action of (−) epicatechin in nociception models in vivo.

Previous experiments conducted in our laboratory, demonstrated this analgesic effect of EPI in different chemical models of nociception, in models of capsaicin, writhing induced by acetic acid, formalin and glutamate in mice. However, the mechanisms involved in this effect of EPI are not known. On this basis, the objective of this study was to evaluate the possible mechanisms involved in the antinociceptive action of EPI in a model of chemical nociception.

## Method

### Aminals

Male Swiss mice (25-30 g) were kept at a temperature of 25 °C in a light/dark cycle of 12 hours with food and water ad libitum. Food was withdrawn 12 hours before the experiments. The animals were taken to the laboratory 2 hours before the experiments in order to adapt to the environment. The protocols were approved by the Ethics in Research Committee of the Federal University of Piauí - UFPI (No. 011/2008). The experiments were performed after approval of the protocol by the Institutional Ethics Committee and were carried out in accordance with the National Institutes of Health Guide for Care and Use of Laboratory Animals (Publication No. 85–23, revised 1985) and the Ethical Guidelines for Investigations of Experimental Pain in Conscious Animals (Zimmermann, 1983). The numbers of animals and intensities of noxious stimuli used were the minimum necessary to demonstrate the consistent effects of the drug treatments. The tests took place at the Medicinal Plants Research Center (NPPM), UFPI.

### Obtaining the plant

The *Combretum leprosum* was collected from the premises of the Centre for Agrarian Sciences, Federal University of Piauí (UFPI) during September 2007 (5°02'57.28"S, 42°46'43.27" W, 93 meters above sea level). After collection, the species was identified and deposited at the Graziela Barroso Herbarium and numbered 011/2008 at the UFPI.

### Drugs

The experiments used: naloxone (opioid antagonist, 2 mg/kg s.c. - Sigma, USA), glibenclamide (potassium channel antagonist-sensitive to ATP, 2 mg/kg s.c. - Sigma, USA), caffeine (adenosine antagonist, 3 mg/kg s.c.), ondansetron (5 HT3 antagonist, 0.3 mg/kg s.c. - Sigma, USA) pindolol (5 HT1 antagonist - beta adrenergic 0.1 mg/kg s.c. - Sigma, USA), ketanserin (5HT _2A_ antagonist, 0.3 mg/kg s.c.), atropine (muscarinic antagonist, 0.01 mg/kg s.c. - Sigma, USA), morphine (opioid agonist 2.5 or 5 mg/kg s.c. - Cristália, BRA), MK 801 (glutamate receptor antagonist, 0.03 mg/kg i.p. - Sigma, USA), L-arginine (precursor of nitric oxide 600 mg/kg i.p. - Sigma, USA), L-nitro arginine (an inhibitor of nitric oxide synthesis, 75 mg/kg i.p. - Sigma, USA), glutamate (excitatory amino acid, 20 mol/paw, Sigma, USA) and pindolol (beta-adrenergic and 5HT1 antagonist, 0.15 mg/kg s.c.). All substances used were dissolved in saline except for the EPI, which was dissolved in saline and dimethyl sulfoxide (DMSO 1 %) and used orally (p.o.). All protocols were carried out 60 min after the administration of EPI, which according to the literature, coincides with the peak plasmatic concentration of this substance [11].

### Obtaining the epicatechin

Dried and powdered barks (1196 g) of *C. leprosum* were extracted six times by maceration with ethanol at room temperature (123 g, 10.3 %). The solvent was removed by evaporation under reduced pressure and part of the extract (95 g) was suspended in a mixture of H_2_O/MeOH (2:1) and subjected to partition with ethyl acetate afforded H_2_O/MeOH (27.0 g, 28.4 %) and EtAcO fractions. The latter was concentrated and suspended in MeOH/H_2_O (9:1), extracted with hexane, providing the hexane (7.0 g, 7.4 %), and hydroalcoholic (52.0 g, 54.7 %) fractions. Part of the hydroalcoholic fractions (11 g) was applied on a column (4.5 X 55 cm) of silica gel eluted with CHCl_3_/MeOH, in order to increase polarity, giving 139 fractions (125 ml each). Fraction G (56–79, 438 mg, CHCl_3_/MeOH 9:1) was applied to a RP-18 column (10 g) using a H_2_O/MeOH (6:4) solvent system to afford G2 (315 mg). This fraction was re-purified on a column of Sephadex LH-20, eluted with MeOH (100 %) to yield (−)-epicatechin (135 mg), identified by ^1^ H and ^13^ C NMR spectroscopic analyses and comparison with the values in the literature (Galotta, et al., 2008). Absolute configuration of the epicatechin was determined by circular dichroism. The spectrum data and chemical structure of the chemical compound are as follows: (−) Epicatechin. Amorphous solid (135 mg, 0.14 %). CD: Δϵ_220–240_ +26.20, Δϵ_280_ -0.52. RMN ^1^ H [300 MHz, CD_3_OD, δ (ppm)]: 4.81 (br s, H-2); 4.18 (br s, H-3); 2.74 (dd; *J* = 16.5 e 3.0 Hz; H-4*anti*); 2.86 (dd; *J* = 16.5 e 4.5 Hz; H-4*syn*); 5.96 (d; *J* = 2.0 Hz; H-6); 5.90 (d; *J* = 2.0 Hz; H-8); 6.98 (d, *J* = 2.0 Hz; H-2’); 6.76 (d; *J* = 8.0 Hz; H-5’); 6.80 (dd; *J* = 2.0 e 8.0 Hz; H-6’). RMN ^13^ C [75 MHz, CD_3_OD, δ (ppm)]: 79.9 (C-2); 67.5 (C-3); 29.2 (C-4); 157.6 (C-5); 95.9 (C-6); 158.0 (C-7); 96.4 (C-8); 157.3 (C-9); 100.1 (C-10); 132.3 (C-1’); 115.3 (C-2’); 145.9 (C-3’); 145.8 (C-4’); 115.9 (C-5’); 119.4 (C-6’).

In summary, the ethanol extract was fractionated in an aqueous phase and an ethyl acetate phase, which in turn was fractionated in the hexane and hydroalcohol phases where the (−) epicatechin was obtained for use in the chemical nociception protocols.

### Assessment of the mechanism of EPI’s antinociceptive activity

The mechanisms involved in the antinociceptive activity of EPI were investigated in the glutamate test, a model of chemical nociception. The dose of EPI used was obtained by constructing dose curves that were previously carried out at our laboratory. The lowest effective dose of EPI was used. The doses of controls were determined according to previous experiments in our laboratory. The control groups received the vehicle (0.1 ml/10 g) used for the dissolution of EPI administered orally (p.o.) or MK 801 0.03 mg/kg administered intraperitoneally (i.p.).

The glutamate test was carried out according to Beirith et al., (1998). The animals (n = 6–8) were treated with EPI (50 mg/kg p.o.) or vehicle (0.1 mL/10 g) 60 min before the intra-plantar (i.pL.) glutamate (20 μmol/paw) injection. The time (s) of licking or biting the paw that received the stimulation for 15 minutes was quantified. The MK 801 (0.03 mg/kg i.p.) was administered 30 min before stimulation and used as a positive control. For the analysis of possible mechanisms of EPI activity in different systems, the animals were pretreated with different antagonists.

The antagonists were used subcutaneously 20 minutes before EPI administration. In order to evaluate the participation of the opioid system, potassium channels sensitive to ATP, the serotoninergic, muscarinic, puri nergic system and nitric oxide pathways, the animals received naloxone (2 mg/kg), glybeclamide (2 mg/kg), ondansetron (0.5 mg/kg), ketanserin (0.3 mg/kg) and pindolol (1 mg/kg) yoimbina (1 mg/kg), atropine (0.01 mg/kg), caffeine (3 mg/kg) and L-arginine (600 mg/kg), respectively.

### Assessment of impairment of motor activity from EPI

The Open Field (30x30x15) and Rotarod tests were carried out to eliminate the interference of the muscle relaxant or central depressant effect on the antinociceptive activity of EPI, observing the number of quadrants invaded during 5 min and the length of time on a rotating bar at 12 rpm, 60 minutes after administration of EPI (50 mg/kg p.o.), diazepam (4 mg/kg i.p.) or vehicle (0.1 mL/10 g v.o.). In the Rotarod test, the maximum time of permanence on the rotating bar was 60 seconds. The animals were screened 24 hours before the experiment, and we selected those who managed to stay for 2 periods of 60 min on the swivel bar.

### Statistical analysis

The results were presented as mean ± SEM. The GraphPad 5.0 software was used. Statistical analysis was carried out using ANOVA (one or two-way) and the Bonferroni test was used as a post-hoc test. The significance level was p <0.05.

## Results

### Analysis of the possible mechanism of EPI’s antinociceptive activity

**Figure 2:**
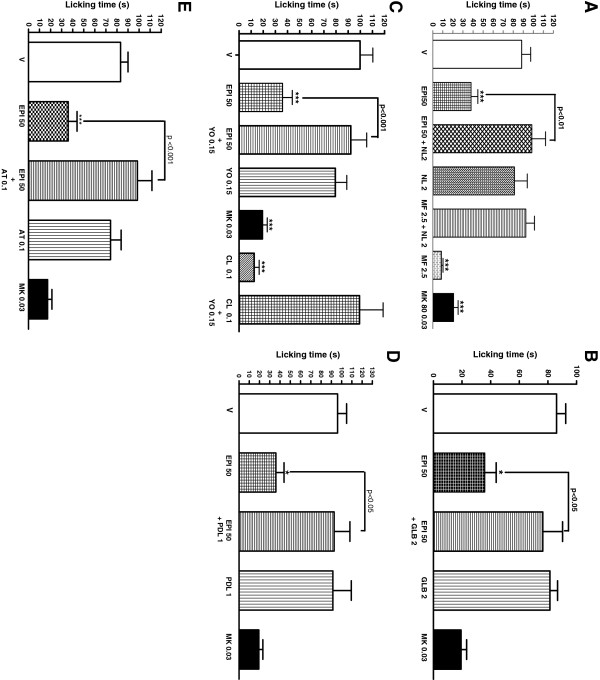
** Possible mechanisms of EPI.** Effect of pretreatment of animals with naloxone (2 mg/kg s.c Figure 2A), glibenclamide (2 mg/kg s.c Figure 2B), yoimbine (0,15 mg/kg s.c Figure 2C), pindolol (1 mg/kg s.c Figure 2D) e atropine (0,2 mg/kg s.c Figure 2E) on the antinociceptive profiles of the (−) epicatequin (EPI) administered orally against glutamate-induced nociception in mice. Each column represents the mean ± S.E.M. of 6 animals. Control value (C) indicates the animals treated with vehicle and the asterisks denote the significance levels, when compared with control groups (one-way ANOVA followed by Bonferroni test) (*P < 0,005; **p < 0,01 e ***p < 0,001).

The pretreatment of animals with the antagonists naloxone (98.63 ± 13.67, p < 0,001), glibenclamide (76.43 ± 13.80, p < 0,05), yoimbina (92.10 ± 13.10, p < 0,001), pindolol (92.68 ± 15.93, p < 0,05) and atropine (98.45 ± 13.07, p < 0,05) completely reversed the antinociceptive effect of EPI in the glutamate test (Figure 2A – 2E). However, there was no reversal when the animals that received the EPI were pretreated with the antagonists ondansetron, ketanserin and L-Arginine and caffeine (The data are not shown). The MK presented a significant antinociceptive effect in the protocols used.

### Effect of EPI on the impairment of motor activity

The pretreatment of animals with EPI did not result in reduction of motor activity in the Open Field (73.85 ± 7.30) and Rotarod (50.54 ± 4.50) models when compared with the vehicle (71.42 ± 8.74 and 56.32 ± 2.11 respectively). Only diazepam (4 mg/kg) caused a significant reduction in motor activity when compared with control.

## Discussion

EPI is primarily known for its antioxidant properties and several studies have evaluated the use of this substance in the treatment of cardiovascular diseases and as a cancer preventive. The possible antinociceptive action of EPI has been demonstrated since it has the ability to block sodium channels, structures that are closely linked to the activation of sensory fibers [8]. The importance of our study is that it investigates the mechanisms involved in the antinociceptive action of EPI, contributes to the knowledge about the properties of *C. leprosum* and its components, and may enable the discovery of new therapeutic options for the treatment of pain.

The activities of EPI were evaluated in a model of glutamate-induced nociception. Glutamate promotes the activation of NMDA receptors, causing an increased influx of calcium with the activation of neuronal NO synthase and nitric oxide formation. Thus, glutamate participates in the processes involved in the perception of and central sensitization to pain [12,13]. According to studies, NO activates the formation of cyclic GMP in the central and peripheral nervous system [14,15].

Thus, blocking these receptors or decrease the release of glutamate would lead to a decrease in pain perception. This effect was observed after the administration of EPI. This blockage also justifies the results found in other models such as capsaicin and formalin, because previous studies show that there is an increased level of glutamate due to the stimulation of C fibers after application of capsaicin or formalin into the paw [16-18].

An important result from our study was demonstrating the involvement of opioid receptors in the antinociceptive effect of EPI. The activation of central and peripheral opioid receptors (mu and kappa mostly) is linked to the inhibition of Ca^++^ currents and activation of K^+^ currents through ATP-dependent channels via activation of Gi. Moreover, these receptors reduce the activation of Na ^+^ channels and TRPV receptors, which also contributes to the antinociceptive response of opioid drugs [19].

In our study, we observed that the antinociceptive action of EPI is reversed by naloxone antagonism, which confirms the possible activation of opioid receptors by EPI. This effect was also reversed by the activity of glibenclamide, an antagonist of K + channels sensitive to ATP, demonstrating the involvement of activation of these channels by EPI. This action was directly related to the inhibition of nociceptive transmission.

Numerous studies show the importance of serotonin (5-HT) in the regulating pathways and the inhibition of nociception. This activity occurs through different receptors (5-HT_1_, 5-HT_2_, 5-HT_3_ and 5-HT_4_) where activation of the receptors 5HT_1_, 5HT_2_ and 5HT_3_ decreases the nociceptive transmission in models such as the formalin one [6,18,20,21]. The results show that the antinociceptive effect of EPI involves the participation of 5HT_2A_ receptors, because we observed a reversal of this effect when animals were pretreated with ketanserin or pindolol, an antagonist of 5HT_2A_ and 5HT_1A_, however, this reversal has not been verified by pre-treatment with ondansetron, which shows that there is no involvement of 5HT_3_ receptors.

In turn, the serotonin system is related to the adrenergic system. The activation of 5-HT receptors may cause an increased release of norepinephrine, which activates the post synaptic alpha 2 receptor and leads to an inhibition of painful impulse conduction. The activity of antagonists such as yoimbina leads to a decreased analgesic effect of the agonists of these receptors, for example, clonidine [22,23]. On this basis, we suggest that EPI presents its antinocicieptive effects through the activation of alpha 2 receptors because the pretreatment of animals with yoimbina reversed its effects in the glutamate model. The activation of alpha 2 receptors could also lead to a decreased release of glutamate in afferent fibers and this would further increase the action of EPI in the studied model [24].

Likewise, the activation of these pathways activate spinal cholinergic interneurons releasing acetylcholine (ACh), which presents an antinociceptive effect probably due to the inhibition of glutamate release as well as the release of GABA from pre-synaptic fibers by the activation of muscarinic receptors antagonized by atropine [25]. Thus, a possible cholinergic agonist activity of EPI would partly justify its antinociceptive effects, because these were reversed by the atropine that was previously administered to the mice in this model.

**Figure 3:**
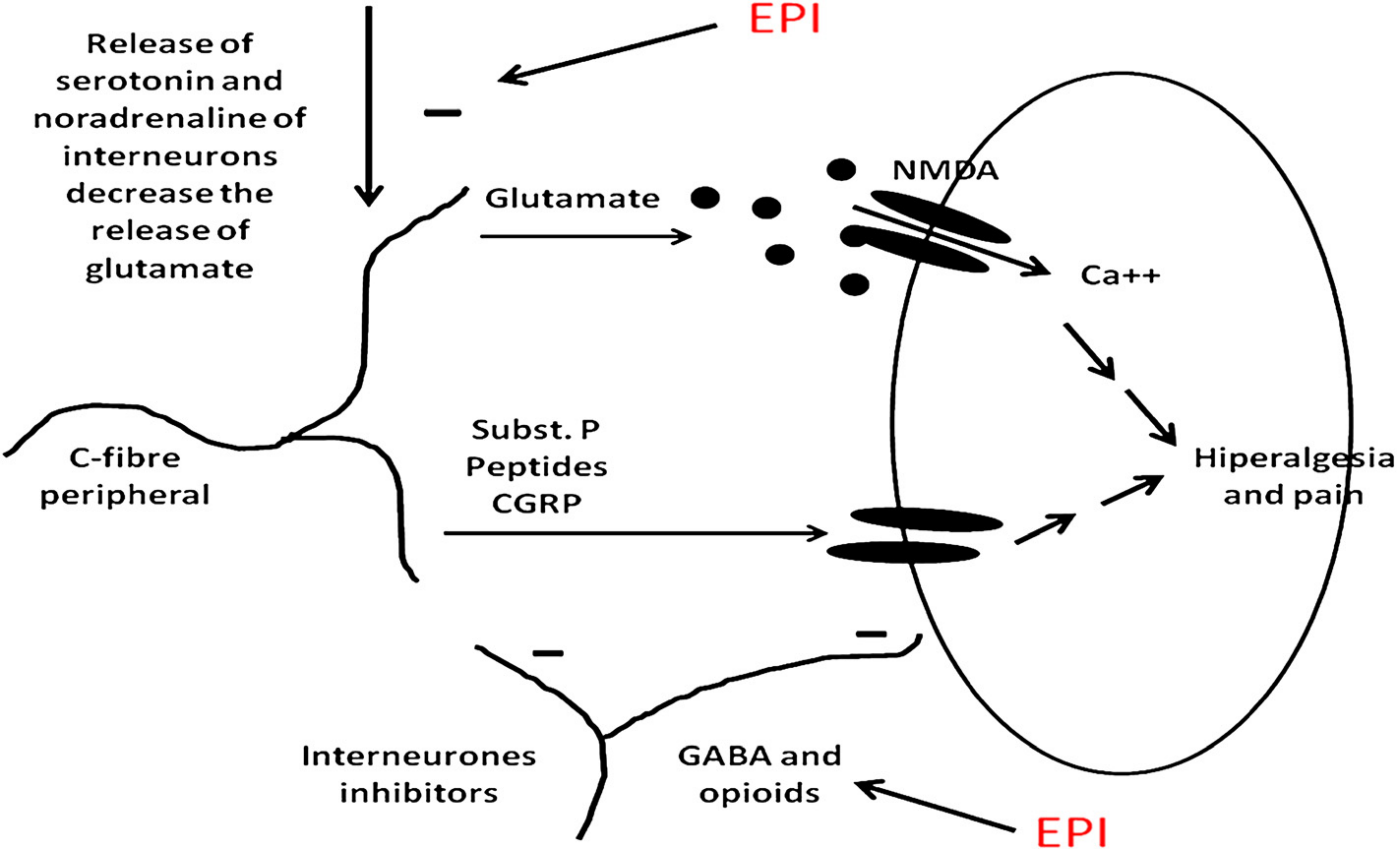
** Possible mechanisms of EPI.** The EPI could act through direct action on serotonin receptors, opioids, acetylcholine or noradrenaline. However, this action might be indirect through the inhibition of glutamate release.

The activation of adenosine receptors (A1) is also important in modulating pain. Agonists of these receptors have significant antinociceptive effect that can be reversed by caffeine or 8-phenyltheophylline [26]. However, this mechanism does not participate in EPI's activities, because pre-treatment with this antagonist did not reverse its effects. Another important pathway in the modulation of nociception is the pathway for nitric oxide. (NO) and cyclic guanosine monophosphate (cGMP). The nitric oxide synthase enzyme may be activated by NMDA glutamate receptors, which leads to the formation of NO that is directly related to the maintenance of nociception [13]. To assess the possible interference of EPI in the formation of NO, animals were pretreated with L-arginine, a precursor of this substance. According to the results, the antinociceptive effect of EPI does not involve the NO/cGMP system as there was no reversal of the effect in animals pretreated with L-arginine when compared with the vehicle. The Figure 3, shows the possible sites of action of EPI.

## Conclusions

In conclusion, this study demonstrates that the antinociceptive activity of EPI in the glutamate model involves the participation of the opioid system with potassium channels sensitive to ATP, serotonin, adrenergic and cholinergic.

## Competing interests

The authors declare that they have no competing interests.

## Authors’ contributions

LSL, R BM, HBF, SSP carried out the studies of pain of mice and drafted the manuscript; MCCA and MHC isolated the (-)EPI of *Combretum leprosum* and FRCA was the leader of the project. All authors read and approved the final manuscript.
